# Advancing periodontitis microbiome research: integrating design, analysis, and technology

**DOI:** 10.3389/fcimb.2025.1616250

**Published:** 2025-06-23

**Authors:** Yibing Han, Pei-Hui Ding

**Affiliations:** Stomatology Hospital, School of Stomatology, Zhejiang University School of Medicine, Zhejiang Provincial Clinical Research Center for Oral Diseases, Key Laboratory of Oral Biomedical Research of Zhejiang Province, Cancer Center of Zhejiang University, Hangzhou, Zhejiang, China

**Keywords:** periodontitis, microbiome, 16S rRNA, metagenome, metatranscriptome

## Abstract

Periodontitis, a chronic inflammatory disease affecting 20%-50% of adults worldwide, is driven by polymicrobial synergy and dysbiosis. Despite numerous studies on the oral microbiota in periodontitis, significant heterogeneity exists between findings, posing challenges for treatment strategies. To understand the sources of this variability and establish standardized protocols, we reviewed the literature to identify potential factors contributing to these discrepancies. We found most studies focus on microbial communities in periodontal pockets, with fewer investigating microbial composition within gingival tissue. Research indicates that bacterial communities in gingival tissue exist as biofilms, potentially serving as reservoirs for persistent infection. Therefore, further exploration of the microbiome within periodontal tissues is needed, which may offer new insights for treatment strategies. Metatranscriptomics provides valuable insights into gene expression patterns of the oral microbiota, enabling the exploration of microbial activity at a functional level. Previous studies revealed that most upregulated virulence factors in periodontitis originate from species not traditionally considered major periodontal pathogens. However, current studies have not fully identified or revealed the functional changes in key symbiotic microbes in periodontitis. We reviewed the analytical paradigms of metatranscriptomics and found that current analysis is largely limited to assessing functional changes in known periodontal pathogens, highlighting the need for a functional-driven approach. Beyond the limitations of current analytical paradigms, the metatranscriptomics also has inherent constraints. We suggested integrating emerging high-throughput microbial sequencing technologies with functional-driven analytical strategies to provide a more comprehensive and higher-resolution insight for microbiome reconstruction in periodontitis.

## 1 Introduction

Affecting an estimated 20% to 50% of the global adult population, periodontitis has long been associated with shifts in the composition and activity of the oral microbiota. Although high-throughput sequencing technologies have deepened our understanding of microbial composition in disease, findings remain highly heterogeneous across studies, hindering the development of unified diagnostic and therapeutic strategies. While most current research focuses on characterizing microbial communities in periodontal pockets, we often overlook the microbial ecosystems embedded within gingival tissues. Recent evidence suggests that these tissue-associated microbes may exist as biofilms and serve as reservoirs for persistent inflammation and treatment resistance. This underexplored niche may hold key insights into disease chronicity and recurrence.

In parallel, metatranscriptomic analyses have offered functional views into microbial gene expression, enabling the identification of active metabolic pathways and virulence factors in periodontitis. Notably, most upregulated virulence factors in periodontitis have been traced to species not traditionally classified as major pathogens, suggesting symbiotics may undergo functional shifts in periodontitis and contribute to disease progression. However, existing metatranscriptomic analyses often prioritize known periodontal pathogens and apply limited functional paradigms, leaving symbiotic and less characterized taxa underexplored.

Here, we review potential factors contributing to the heterogeneity of microbial composition observed across studies, aiming to support a more comparable and structured research framework. We also revisit recent findings on gingival tissue-associated microbiota and present emerging tools that reveal high-resolution distribution of intracellular microbes, facilitating more accurate and comprehensive analyses of host–microbe interactions. On the functional side, we critically assess how current metatranscriptomic approaches—limited by analytical biases and technology constraints—fail to capture the full spectrum of microbial contributors to disease. Collectively, this perspective outlines the conceptual and technical challenges impeding current microbial research in periodontitis, and to advocate for a paradigm shift toward more comprehensive, high-resolution, comparable approaches and the integration of advanced technologies.

## 2 Compositional changes in microbiota during periodontitis

### 2.1 Microbiota in periodontal pockets

Numerous studies have employed 16S rRNA gene sequencing and metagenomic approaches to compare the subgingival microbial composition between healthy individuals and those with periodontitis. While these studies have consistently implicated certain classic periodontal pathogens, the changes in other microbial taxa remain inconsistent across investigations. Here, we focus on the subgingival microbial shifts associated with chronic periodontitis compared to health, reviewing studies employing 16S rRNA gene sequencing or metagenomics to compare the bacteriome in periodontally healthy human subjects and subjects diagnosed with chronic periodontitis within the same study. (Abusleme et al., 2013; Altabtbaei et al., 2021; Belstrøm et al., 2021; Griffen et al., 2012; Kirst et al., 2015; Koohi-Moghadam et al., 2024; Kumar et al., 2005; Li et al., 2014; Liu et al., 2012; Pei et al., 2020; Pérez-Chaparro et al., 2018; Shi et al., 2018; Tsai et al., 2018) (**Supplementary Table 1**). Multiple factors likely contribute to the observed heterogeneity, including demographic variables, general health status, definitions of periodontitis and health, and sampling methodologies. Host genetic predisposition represents a potent but undercharacterized determinant of microbial variation. While significant environmental factors explain 6.36%–7.78% of the β-diversity variance in the oral microbiome, an equivalent number of host SNPs account for 10.14%–14.14% of the variance (Liu et al., 2021). This genetic modulation may contribute to the observed heterogeneity, which appears to reflect distinct host-microbe interaction patterns rather than methodological artifacts alone. Addressing these sources of variability will be critical to establishing reliable microbial changes associated with periodontitis and their role in disease pathogenesis.

Geographically, research on subgingival microbiome composition is heavily skewed, with five studies conducted in the United States, five in China, and the remainder distributed across Danmark, Brazil, and Chile. This geographic bias limits global periodontal microbiome insights. While geographic variability inherently reflects ethnic differences, few studies documented ethnic parameters. Beyond geographic and ethnic variability, inconsistent age stratification further complicates comparisons. While most studies have implemented age-based inclusion criteria, the selected age ranges vary significantly across studies, with some cohorts showing no overlap. The influence of age on the composition of the oral microbiome remains contested. Sarafidou et al. proposed a bidirectional relationship between aging and the oral microbiome (Sarafidou et al., 2024), whereas Feres et al. argued that aging does not substantially alter the composition of the oral microbiome (Feres et al., 2016). This discordance underscores the need for standardized age stratification and longitudinal assessments in future study, as these could clarify the relationship between age and the oral microbiome, as well as the reliable relationship between periodontitis and the oral microbiome.

The lack of consensus on the minimum duration between antibiotic exposure and enrollment in oral microbiome studies complicates the research (Zaura et al., 2021). Among the reviewed studies, seven excluded individuals who had used antibiotics within three months prior to sampling, while four studies extended this exclusion period to six months. Future research should design longitudinal cohorts to track the recovery of the oral microbiome in individuals with antibiotic exposure over different timeframes, thereby providing clearer exclusion criteria.

The criteria used to define periodontal health and periodontitis vary considerably across studies, leading to inconsistencies in patient stratification. To address this issue, we recommend adopting the latest guidelines for defining periodontitis and recording all clinical parameters in detail.

Standardization of sampling protocols is critical, as methodological variations significantly impact microbiome profiles (Smith et al., 2024). Key steps include pre-sampling procedures, sampling tools selection and sampling sites selection. In most studies, supragingival plaque is removed prior to sampling subgingival plaque. Additionally, three studies delayed sample collection by one week following periodontal examination. This approach aims to reduce the impact of bacterial translocation caused by periodontal probing (Altabtbaei et al., 2021). Where feasible, this strategy is recommended to enhance the accuracy and reliability of microbial data. For sampling tools, most studies utilize curettes or paper points. The curetting method primarily collects attached plaque from the surfaces of teeth, whereas the adsorption method using paper points mainly capturing unattached bacteria within periodontal pockets (Lu et al., 2022). However, the impact of these sampling tools on the observed composition of the subgingival microbiome remains poorly understood and warrants future research.

The selection of sampling sites is mainly guided by two key considerations: (1) the distribution of teeth and anatomical sites (e.g., mesial, distal, buccal, lingual), or (2) clinical parameters falling within a defined range. Most studies tended to focus on just one of these two aspects. Focusing on tooth distribution and specific surfaces aims to ensure the representativeness of the overall periodontal condition while minimizing saliva contamination. Previous study recommended selecting Ramfjord index teeth (Ramfjord, 1959) to represent overall periodontal condition and the mesial and distal buccal sides of the target teeth (Lu et al., 2022) to avoid saliva contamination as possible. However, among the reviewed studies, none employed Ramfjord index teeth in their sampling protocols, and only two studies selected mesial-buccal sites. Two studies included teeth from all four quadrants. While this approach aims to improve sampling representativeness, its representativeness of the overall periodontal condition remains unclear and warrants further investigation.

Selecting sampling sites using clinical parameters is reliant upon an assumption: different clinical parameters may correspond to distinct microbial communities. However, most studies (Ge et al., 2013; Kumar et al., 2005; Pérez-Chaparro et al., 2018; Shi et al., 2018; Wirth et al., 2021) suggest that microbial structure in the same patient was similar between periodontal pockets of different depth (**Supplementary Table 2**). Despite these insights, site-level studies remain limited in number and have exclusively relied on 16S sequencing to describe microbial taxonomy. Moreover, these studies have predominantly focused on a single clinical parameter—probing depth—while employing inconsistent probing depth thresholds to define pocket categories. Hence, future research should expand its scope to include a broader range of clinical parameters and analyze their influence to oral microbiome.

The heterogeneity of microbial composition in periodontitis may not stem from confounding factors but rather reflect inherent patient stratification. This concept parallels findings in gut microbiome research (MetaHIT Consortium (additional members) et al., 2011), where the integration of 39 sequenced fecal metagenomes from 6 nationalities revealed three robust clusters (enterotypes), each characterized by distinct species composition and functional profiles. In contrast, the oral microbiome field lacks large-scale, integrated meta-analyses of high-quality data. Such efforts may uncover analogous microbial signatures in periodontitis, potentially identifying distinct “periodontotypes” that reflect underlying biological variations. By delineating these subgroups, researchers could pave the way for precision medicine approaches tailored to specific microbial profiles, ultimately improving diagnostic accuracy and therapeutic outcomes in periodontitis.

### 2.2 Microbiota within gingival tissue

Distinct microbial profiles were observed between periodontal pockets and gingival tissues, with marked differences in bacterial loads, diversity, and community structure. Total bacterial loads in periodontal pockets were 1 to 4 orders of magnitude higher than those within gingival tissues, reflecting a stark contrast in microbial density between these niches (Baek et al., 2018). The microbial composition was more consistent across samples within gingival tissues than samples in periodontal pockets, suggesting a more uniform microbial environment within the gingival tissue than in periodontal pockets (Baek et al., 2018). Additionally, in gingival tissue, a highly specialized microbial community is evident, with only 14 species accounting for over 80% of 16S rRNA gene reads (Bao et al., 2020).

At the genus level, *Fusobacterium*, *Porphyromonas*, *Actinobaculum*, and *GG703879_g* (Actinomycetaceae family) were significantly enriched in gingival tissues, while *Bulleidia*, GQ422727_g (Peptococcaceae family), and *Coriobacteriaceae_uc* were reduced (Baek et al., 2018). Among these, *Fusobacterium nucleatum* and *Porphyromonas gingivalis* were particularly enriched in gingival tissue and exhibited a coexistence pattern mainly in areas of inflammatory infiltration, suggesting a potential role in causing tissue inflammation (Baek et al., 2018). However, the mechanistic underpinnings of how these intratissue microbial communities differ from their periodontal pocket counterparts remain poorly understood and warrant further investigation.

Biofilm formation within gingival tissues was further observed using alcian blue staining and atomic force microscopy (AFM) (Baek et al., 2018). The degree of biofilm formation did not always depend on bacterial density but was associated with the extent of tissue destruction (Baek et al., 2018). This observation implies that biofilm architecture, beyond mere bacterial load, may play a critical role in facilitating immune evasion and sustaining chronic inflammation. Current periodontal treatments primarily focus on removal of subgingival dental plaque in periodontal pockets. However, the presence of biofilms within gingival tissues challenges the conventional paradigm and highlights the need to reconsider tissue-invasive bacteria as critical players in periodontitis. Moreover, the presence of biofilms within gingival tissues suggests the need for therapeutic delivery strategies capable of penetrating gingival tissue and disrupting intra-tissue microbial communities, rather than focusing solely on surface-level biofilm removal. Such a shift could refine our understanding of periodontal disease pathogenesis, uncover novel therapeutic targets, and ultimately lead to more effective treatment strategies that complement existing approaches.

Beyond the microbial differences between periodontal pockets and gingival tissues in periodontitis, the gingival tissue microbiome in periodontitis also shifts significantly compared to healthy conditions (Bao et al., 2020). In healthy tissues, *Streptococcus vestibularis* dominates, followed by *Haemophilus parahaemolyticus* and *Veillonella dispar*. These species likely contribute to biofilm structural integrity and tissue barrier maintenance without provoking inflammation (Kolenbrander, 2011; Teles et al., 2012). Diseased tissues are characterized by the dominance of an as-yet-uncultured species, *Treponema* sp. Human Microbial Taxon (HMT) 253. 11 operational taxonomic units (OTUs) significantly differentiate health from disease, including five from the 14 most abundant species: *S. vestibularis*, *Treponema* sp. HMT 253, *V. dispar*, *Fusobacterium naviforme*, and *Selenomonas* sp. HMT 478. This suggests that both high- and low-abundance species collectively contribute to the microbial signature of gingival tissue microbiome in periodontitis.

The “Single-cell Analysis of Host-Microbiome Interactions” (SAHMI) pipeline provided a powerful solution for studying microbial distribution at single-cell resolution by identifying sparse bacterial reads from single-cell datasets (Ghaddar et al., 2022). Analysis of healthy and diseased keratinocytes revealed that 0.5-2% of all barcodes harbored at least one bacterial read (Easter et al., 2024). While healthy samples showed few cell-microbial associations, diseased samples exhibited significantly higher bacterial reads per cell, highlighting an increase in microbial invasion during disease progression (Easter et al., 2024). Among the detected species, *Porphyromonas gingivalis* exhibited the most dramatic increase, with a nearly 200-fold enrichment in keratinocytes and immune cell populations (Easter et al., 2024). *Treponema vincentii* displayed a different upregulation pattern, primarily enriched in lymphatic endothelial cells and immune cells (Easter et al., 2024). This cell-specific enrichment suggests that different pathogens may exploit unique niches within the gingival tissue, potentially contributing to diverse pathogenic mechanisms during disease progression. These findings underscore the importance of studying both bacterial tropism and host cell specificity to better understand the complex interplay between microbes and the host in periodontitis.

While the SAHMI pipeline provides valuable insights, it has notable limitations. Nonspecific RNA capture may skew the detection rates of specific taxa, potentially altering overall microbial profiles. The INVADEseq approach overcomes this limitation. Built on the backbone of the 5′ Chromium scRNA assay, INVADEseq simultaneously primes bacterial 16S rRNA and host mRNA within single-cell Gel Bead-in-Emulsions (GEMs) (Galeano Niño et al., 2022, 2023). This innovative method enables the analysis of host-bacterial interactions directly at single-cell resolution, offering a more robust and accurate platform for studying microbial distribution in complex tissues.

Notably, it still remains unclear whether all detected bacteria were true tissue invaders or merely superficial persisters that survived the washing steps. Hence, the oral microbiome data obtained from gingival tissues only identified potentially invasive species of the periodontium.

## 3 Functional changes in microbiota during periodontitis

In periodontitis, changes in microbial abundance do not always correspond to functional activity (Dabdoub et al., 2016), suggesting that focusing solely on species composition may fail to comprehensively reflect disease status. For example, species such as *Porphyromonas* sp. *274*, *Corynebacterium*, and *Leptotrichia hofstadii*, despite their relatively low abundance, occupy a substantial proportion of metatranscriptomic libraries in periodontitis. Additionally, the greater consistency in microbial functional expression compared to microbial composition (Duran-Pinedo et al., 2014) implies that periodontitis progression depends more on conserved functional activities than on particular microbial composition. Therefore, exploring microbial functional expression is critical for uncovering key mechanisms involved in disease progression.

Current studies (Duran-Pinedo et al., 2014; Jorth et al., 2014; Szafrański et al., 2015; Yost et al., 2015) primarily focus on known periodontal pathogens, such as the red and orange complexes, finding they upregulating their proportion and genes associated with virulence and specific metabolic pathways in periodontitis. Duran-Pinedo et al. (2014) analyzed the changes in virulence factor expression within the red complex and found that *Porphyromonas gingivalis* upregulated the expression of hemolysin genes, vitamin B12 uptake genes in disease, while *Treponema denticola* increased the expression of genes related to oligopeptide transport, flagella synthesis, and vitamin B12 uptake. Szafrański et al. (2015) focused on one of the orange complexes—*Prevotella nigrescens*, which upregulated most genes were associated with peptidases, heme ABC transporters, multidrug transporters, collagenase, hemagglutinin, L-asparaginase, L-aspartate oxidase, fumarate hydratase, acyl-CoA synthetase, and NAD-utilizing dehydrogenase.

However, this reliance on previously reported species may limit the discovery of new pathogenic mechanisms, as certain species traditionally considered commensals may undergo functional shifts in periodontitis and contribute to disease progression. Duran-Pinedo et al. (2014) observed virulence factors upregulated most in *Neisseria*, *Corynebacterium*, *Rothia dentocariosa*, *Veillonella parvula*, and *Actinomyces*—microbes previously associated with periodontal health—without further exploring their functional contributions to disease. Supporting this paradigm, *Streptococcus gordonii*, another commensal organism typically linked to periodontal health, has been shown to promote periodontitis pathogenesis by facilitating *Porphyromonas gingivalis* intracellular survival (Croft et al., 2018) and stimulating IL-8 production in human periodontal ligament cells (Kim et al., 2017). Addressing this gap requires a function-driven perspective that identifies previously overlooked pathogenic species by tracing dysregulated metabolic/virulence pathways back to their microbial executors, thereby revealing novel therapeutic targets beyond traditional periodontal pathogens.

Fully elucidating the microbial drivers of periodontal pathology requires not only improved analytical strategies, but also advances in sequencing technologies. Current studies analyzing functional change predominantly rely on metatranscriptomic sequencing. Metabolomics offers a complementary perspective by capturing downstream biochemical products and providing a snapshot of microbial and host-microbe metabolic interactions. Despite its complementary value, metabolomics is rarely integrated with metatranscriptomics, limiting the capacity to connect microbial transcriptional activity with associated metabolic outputs.

Metatranscriptomics itself also has notable limitations. First, species identification through single-read alignment can be unreliable, and second, metatranscriptomics masks the functional heterogeneity that exists within the same species, may obscuring functionally active subpopulations. Emerging single-cell microbiological technologies provide a transformative solution to these challenges by achieving accurate species-level identification combining multi-read alignment within individual cell and enabling high-resolution transcriptional expression within functionally distinct microbial subpopulations in complex ecosystems (Lloréns-Rico et al., 2022). Notable advances, such as droplet-based platforms like BacDrop (Ma et al., 2023) and smRandom-seq (Xu et al., 2023), have successfully resolved microbial functional heterogeneity in human gut (Shen et al., 2025) and rumen (Jia et al., 2024). However, its application to the relative low abundance oral microbiome presents significant challenges, necessitating further research to optimize these technologies for low-biomass environments. In addition to experimental hurdles, analytical methods for high-dimensional single-cell microbiological sequencing and strategies for multi-omics integration remain limited, highlighting the need for scalable and reproducible computational pipelines. Future research may build upon these advanced single-cell techniques and employ function-driven strategies to uncover novel disease-driving species and elucidate their specific roles in periodontitis progression.

## 4 Discussion

Accumulating evidence has revealed the compositional and functional complexity of the subgingival microbiome in periodontitis. Nonetheless, heterogeneity of microbial composition across studies remains—stemming from demographic diversity, methodological variation, and possibly unrecognized biological subtypes of the disease. Addressing this variability will be critical for identifying robust microbial signatures, improving cross-study comparability for integrative microbiome research.

While periodontal pocket remains the most commonly explored niche, gingival tissue represents a distinct and underexplored component of the periodontal ecosystem. The presence of structured microbiota within gingival tissues—coupled with their association with chronic inflammation—challenges conventional paradigms and highlights these niches as underappreciated contributors to disease persistence and treatment resistance. Understanding both the bacterial tropism and host cell specificity potentially refine our understanding of the complex interplay between microbes and the host in periodontitis.

Functional profiling has revealed that microbial activity in periodontitis often diverges from taxonomic abundance, with low-abundance species may exhibiting disproportionate virulence. Current metatranscriptomic approaches remain limited by analytical paradigm and insufficient resolution. Emerging single-cell microbiological technologies offer a path forward by capturing single cell level activity and uncovering functional heterogeneity within one species. Integrating these tools with function-driven strategies will be critical for identifying overlooked pathogens and advancing precision diagnostics and therapeutics in periodontitis.

Moving forward, we advocate for a more comprehensive and integrated framework for oral microbiome research—one that encompasses both extracellular and intracellular microbial communities, supported by standardized cohort design and sampling strategies, and enabled by advanced sequencing technologies for high-resolution, functionally contextualized analyses. Such a shift will be essential to uncovering microbial signatures that are not only robust and reproducible, but also clinically actionable, enabling precision diagnostics and targeted interventions in periodontal disease.

## Data Availability

The original contributions presented in the study are included in the article/supplementary material. Further inquiries can be directed to the corresponding author.
